# TPMA: A two pointers meta-alignment tool to ensemble different multiple nucleic acid sequence alignments

**DOI:** 10.1371/journal.pcbi.1011988

**Published:** 2024-04-01

**Authors:** Yixiao Zhai, Jiannan Chao, Yizheng Wang, Pinglu Zhang, Furong Tang, Quan Zou

**Affiliations:** 1 Institute of Fundamental and Frontier Sciences, University of Electronic Science and Technology of China, Chengdu, China; 2 Quzhou People’s Hospital, Quzhou Affiliated Hospital of Wenzhou Medical University, Quzhou, China; 3 Yangtze Delta Region Institute (Quzhou), University of Electronic Science and Technology of China, Quzhou, China; 4 Department of Basic Medical Sciences, School of Medicine, Tsinghua University, Beijing, China; KAUST, SAUDI ARABIA

## Abstract

Accurate multiple sequence alignment (MSA) is imperative for the comprehensive analysis of biological sequences. However, a notable challenge arises as no single MSA tool consistently outperforms its counterparts across diverse datasets. Users often have to try multiple MSA tools to achieve optimal alignment results, which can be time-consuming and memory-intensive. While the overall accuracy of certain MSA results may be lower, there could be local regions with the highest alignment scores, prompting researchers to seek a tool capable of merging these locally optimal results from multiple initial alignments into a globally optimal alignment. In this study, we introduce Two Pointers Meta-Alignment (TPMA), a novel tool designed for the integration of nucleic acid sequence alignments. TPMA employs two pointers to partition the initial alignments into blocks containing identical sequence fragments. It selects blocks with the high sum of pairs (SP) scores to concatenate them into an alignment with an overall SP score superior to that of the initial alignments. Through tests on simulated and real datasets, the experimental results consistently demonstrate that TPMA outperforms M-Coffee in terms of aSP, Q, and total column (TC) scores across most datasets. Even in cases where TPMA’s scores are comparable to M-Coffee, TPMA exhibits significantly lower running time and memory consumption. Furthermore, we comprehensively assessed all the MSA tools used in the experiments, considering accuracy, time, and memory consumption. We propose accurate and fast combination strategies for small and large datasets, which streamline the user tool selection process and facilitate large-scale dataset integration. The dataset and source code of TPMA are available on GitHub (https://github.com/malabz/TPMA).

## Author summary

Accurate multiple sequence alignment (MSA) is vital for comprehensive biological sequence analysis. However, as no single MSA tool consistently outperforms others across diverse datasets, researchers must invest significant time exploring multiple tools to identify the most suitable one for their specific dataset. To address this, researchers seek tools that can merge locally optimal results from diverse initial alignments into a globally optimal alignment. Our novel approach, Two Pointers Meta-Alignment (TPMA), employs a two-pointer to partition initial alignments into blocks, selecting those with the higher sum of pairs (SP) scores for integration into a globally optimal alignment. TPMA consistently outperforms M-Coffee, demonstrating superior aSP, Q, and total column (TC) scores, coupled with faster running times and lower memory consumption. We present comprehensive assessments of various MSA tools, proposing efficient combination strategies for diverse datasets. Our tool, TPMA, and associated resources are publicly available on GitHub (https://github.com/malabz/TPMA), offering a valuable contribution to the field of evolutionary biology and streamlining the selection process for users dealing with large-scale datasets.

## Introduction

Multiple sequence alignment (MSA) is a common technique for analyzing biological sequences, encompassing structure and function estimation, evolutionary relationship determination, gene identification, and more [1]. Hence, the precision of MSA directly shapes the outcomes of analyses in bioinformatics, holding paramount importance in discerning biological significance. However, due to its time-consuming nature, many MSA tools, like MAFFT [2] and MUSCLE3 [3], resort to heuristic methods to address computational complexities. Despite their extensive usage, tools employing heuristic methods exhibit two common limitations. Firstly, the persistence of an incorrectly inserted gap, adhering to the "once a gap, always a gap" rule, can influence subsequent alignments. The other issue is the presence of a local optimal trap. According to the greedy principle, the optimal solution for each subproblem may not necessarily be equivalent to the optimal solution for the global problem. To overcome the drawbacks above, researchers typically employ iterative optimization and meta-alignment methods to further enhance alignment accuracy.

Iterative optimization involves refining the existing alignment by realigning potential low-quality regions, employing two partition methods: vertical and horizontal realigners. Refin-Align [4], a vertical realigner, partitions the initial alignment into blocks based on columns with identical bases. It then eliminates gaps within each block and realigns the sequences using Promalign. If the new alignment exhibits higher accuracy, it replaces the initial alignment. On the other hand, the horizontal realigners, including REFINER [5], RF [6], and ReformAlign [7], adopt distinct strategies. Similar to REFINER, RF randomly selects a sequence in each iteration and realigns it with the profile of the remaining sequences; if the accuracy of the new alignment improves, it serves as the input for the next iteration round until the objective function score converges or reaches the cycle limit. ReformAlign differs from the others by independently realigning all sequences within a single iterative round. This realignment is performed using a summarization profile constructed from the initial alignment. When new gaps are inserted into the profile, ReformAlign automatically switches to a profile fine-tuning mode to account for the new insertion(s), and once the profile is successfully updated, the realignment is restarted using the new profile until the new alignment is obtained after all sequences have been realigned. However, among the methods mentioned above, currently only ReformAlign is available.

Meta-alignment, a process aimed at synthesizing multiple initial alignments, is employed to derive a high-accuracy alignment. The ComAlign algorithm [8] identifies qualitatively conservative blocks within the initial alignments and amalgamates them through dynamic programming to produce a novel alignment, often exhibiting improvements. MergeAlign [9] generates a weighted directed acyclic graph (DAG), wherein each node represents the site combinations of each column in the initial alignments. Each edge signifies a transition between two adjacent columns, and the weight of each edge corresponds to the number of initial alignments containing that particular transition (between two adjacent columns). Finally, the nodes (columns) in the maximum weight path form the merged alignment. AQUA [10], an automated quality improvement program for Multiple Sequence Alignment (MSA), initiates its process by generating two initial alignments through MUSCLE3 and MAFFT. These alignments are subsequently independently optimized using RASCAL [11], resulting in two refined alignments. In its culmination, the meta-alignment process yields the alignment with the highest accuracy among the four, determined by the NorMD score. The most widely utilized meta-alignment method is M-Coffee [12], which constructs a consistency library of each pair of residues with residue pairs from all other alignments to increase the weight of the aligned pairs accepted by most MSA tools. However, it is noteworthy that this inclusive approach implies that incorrectly aligned pairs prevalent across multiple alignments will also be weighted. Consequently, the accuracy of the M-Coffee meta-alignment can either be improved or diminished, depending on the accuracy of each individual MSA tool.

In this study, we have developed TPMA, a meta-alignment tool for integrating nucleic acid sequence alignments, which employs a two-pointers approach to partition the initial alignments into blocks with identical sequence fragments and concatenate blocks with higher SP scores to form the final alignment. We tested TPMA, M-Coffee, and ReformAlign on four simulated and six real datasets. The results consistently show that TPMA outperforms M-Coffee in terms of aSP, Q, and TC scores across most datasets. Notably, in cases where TPMA scores closely align with M-Coffee, TPMA exhibits significantly lower time and memory consumption. In datasets with varying similarity spans, ReformAlign optimization led to approximately 50% of alignments experiencing decreased TC scores in simulated datasets. Thus, TPMA exhibits superior performance compared to both M-Coffee and ReformAlign. To save time for users while selecting MSA tools, we screened all MSA tools used in our experiments based on accuracy, time, and memory consumption. Therefore, we propose accurate and fast combination strategies to integrate small and large datasets, ensuring efficient integration while upholding alignment accuracy.

## Results

### TPMA outperforms M-Coffee in alignment quality, running time, and memory consumption

In this experiment, we employed two simulated datasets, 16S-like and 23S-like rRNA, along with four real datasets: 16S rRNA, human mitochondrial (mt) genomes, SARS-CoV-2_20200301, and SARS-CoV-2_20200417 datasets. Furthermore, nine MSA tools, namely ClustalW2, Dialign-TX, Kalign3, MAFFT, MUSCLE3, MUSCLE5, PCMA, POA, and T-Coffee, were employed to generate initial alignments. Detailed information regarding the versions and operating parameters of these MSA tools can be found in S10 Table.

M-Coffee takes the original, unaligned sequences dataset as input and utilizes the "-method" parameter to specify the MSA tools for obtaining the initial alignments. To ensure compatibility with M-Coffee, we crafted a shell script specifically for calling MUSCLE5, while the remaining MSA tools have already been integrated into M-Coffee by default. The shell script for invoking MUSCLE5 and its usage instructions can be found at https://github.com/malabz/TPMA/tree/main/tools. The input for TPMA comprises multiple initial alignments generated by different MSA tools, with each aligner’s running parameters precisely identical to those utilized in the M-Coffee, guaranteeing an impartial experimental setup. Two simulated datasets with references were evaluated by aSP, Q, and TC scores, while the alignment quality of the four real datasets was assessed based on the aSP score. The running time of TPMA refers to the duration taken to merge multiple initial alignments, while memory consumption indicates the peak amount of memory utilized during this merging process. Regarding M-Coffee, the running time and memory consumption encompass the time and maximum memory used from initial library construction to combination completion. Specifically, the reported running time and memory consumption by TPMA are related to the merging process. In the case of M-Coffee, its memory usage during merging corresponds to the peak memory usage of its final output. To measure the timing of the M-Coffee merging process, we introduced a timestamp mechanism. This entailed placing a timestamp at the start of merging, following the if function in line 5093 of the t_coffee.c file in its source code. Another timestamp was logged upon program completion, enabling us to accurately compute the duration of the M-Coffee merging process. The time and memory consumed by the MSA tools generating the initial alignments were excluded for the purpose of comparing TPMA and M-Coffee.

**Fig 1:**
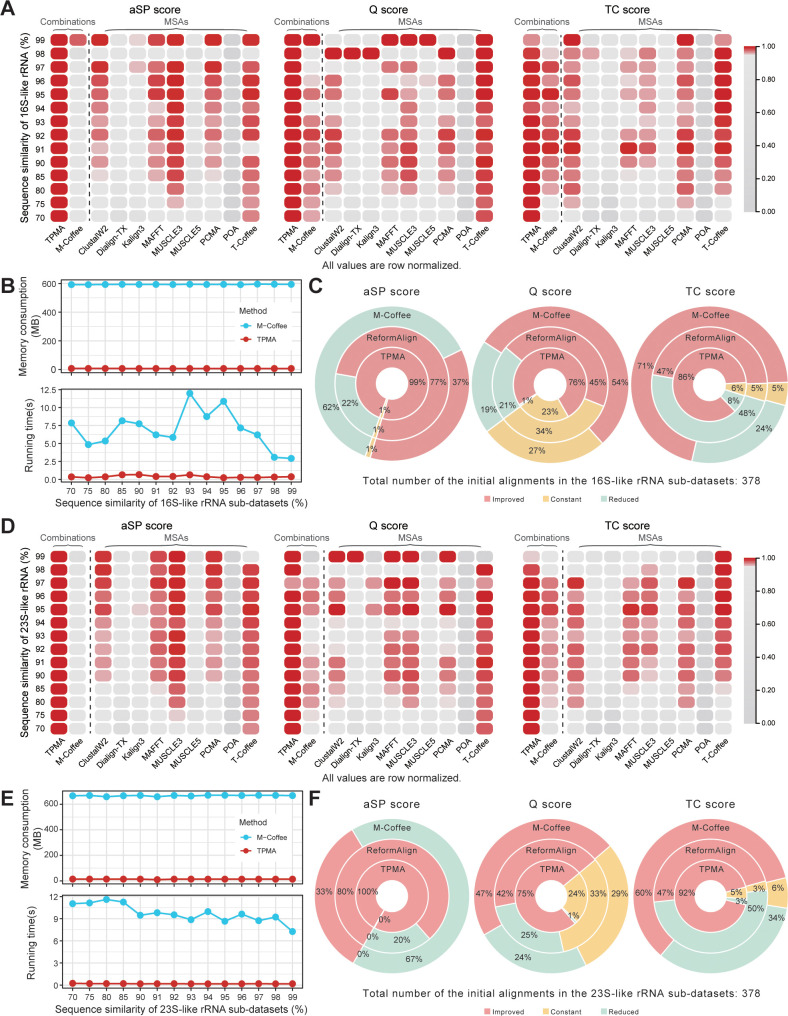
Comparative analysis of TPMA, M-Coffee, and ReformAlign on 16S-like and 23S-like rRNA datasets. Each dataset consists of 14 sub-datasets, each exhibiting varying levels of sequence similarity ranging from 99% to 70%. Within each similarity sub-dataset, three replicates are included. For each replicate, nine initial alignments are acquired and subsequently merged using TPMA and M-Coffee. **A, D** The aSP, Q, and TC scores of TPMA, M-Coffee, and nine MSA tools across 16S-like and 23S-like rRNA datasets. For each gird, the average of three repetitions in one sub-dataset was calculated and subjected to min-max normalization using the alignment scores from all tools. **B**, **E** The changes in running time and memory usage of TPMA and M-Coffee across different levels of similarity for the 16S-like and 23S-like rRNA datasets. Each point represents the average time and memory consumption resulting from the combination of TPMA and M-Coffee across the three replicates. This computation excludes the time and memory required for obtaining the initial alignments. **C, F** Compare improvements in aSP, Q, and TC scores of TPMA, M-Coffee, and ReformAlign for initial alignments on 16S-like and 23S-like rRNA datasets. Each dataset consists of 14 sub-datasets, with three replicates per sub-dataset. A total of 378 (14×3×9) initial alignments were generated using 9 MSA tools. ReformAlign optimized these 378 initial alignments, calculating the differences between the scores of the optimized and unoptimized initial alignments for aSP, Q, and TC scores. Additionally, the disparities between the scores of the merged alignments (through TPMA and M-Coffee) and the unmerged initial alignments were also computed for aSP, Q, and TC scores. A total of 378 differences were obtained for TPMA and M-Coffee, obtained from 14×3 replicates, with each replicate generating 9 differences. The proportions of "improved," "constant," and "reduced" are summarized in donut charts.

**Fig 2:**
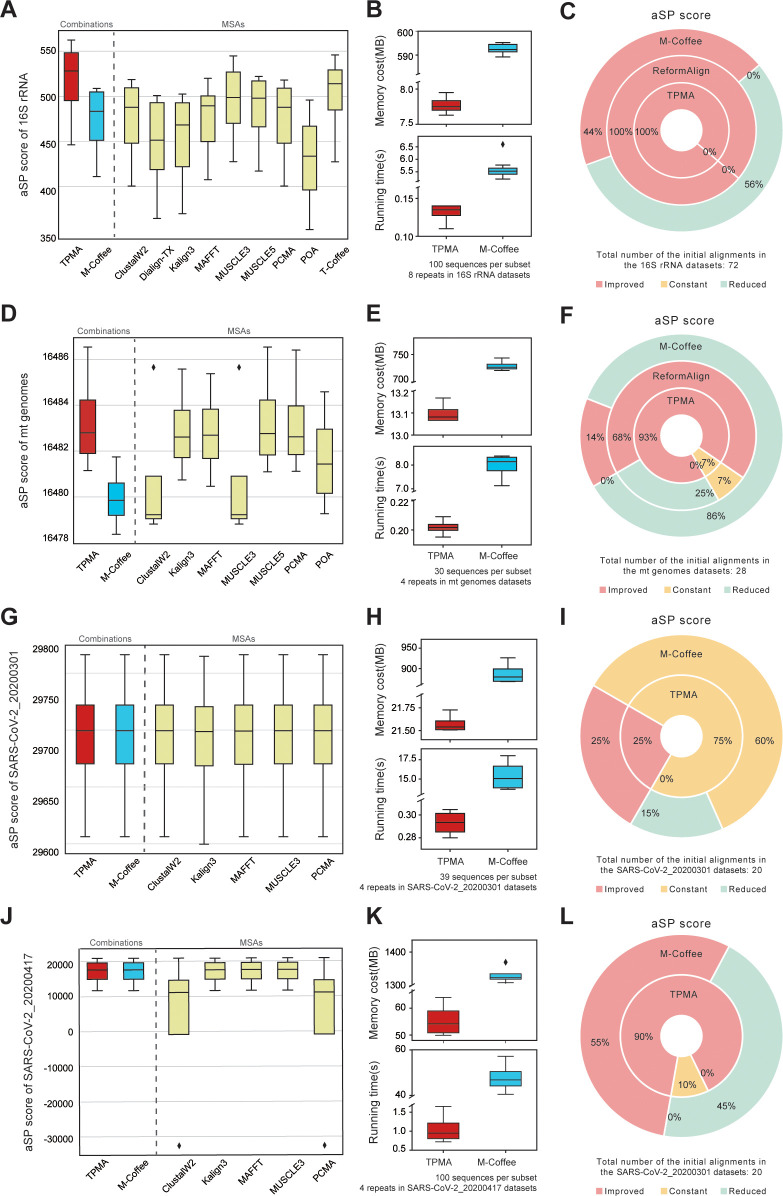
Comparative analysis of TPMA, M-Coffee, and ReformAlign across the four real datasets. The 16S rRNA datasets include 8 subsets, each comprising 100 sequences, and nine MSA tools were utilized to generate the initial alignments for these datasets. Additionally, the mt genomes, SARS-CoV-2_20200301, and SARS-CoV-2_20200417 datasets consist of 4 subsets containing 30, 39, and 100 sequences, respectively. **A, D, G, J** The aSP score of TPMA, M-Coffee, and various MSA tools on the 16S rRNA, mt genomes, SARS-CoV-2_20200301, and SARS-CoV-2_20200417 datasets. It’s worth noting that some MSA tools failed to align on these datasets; only the tools depicted in the figure completed the alignment. The resulting initial alignment was then utilized for the merging process using TPMA and M-Coffee. **B, E, H, K** The running time and memory usage of TPMA and M-Coffee for the 16S rRNA, mt genomes, SARS-CoV-2_20200301, and SARS-CoV-2_20200417 datasets. Notably, the values exclude the time and memory needed to obtain the initial alignments. **C, F, I, L** The enhancements in the aSP score of initial alignments on the 16S rRNA (**C**), mt genomes (**F**), SARS-CoV-2_20200301 (**I**), and SARS-CoV-2_20200417 (**L**) datasets among TPMA, M-Coffee, and ReformAlign. Specifically, the 16S rRNA datasets comprise 72 (8×9) initial alignments, while the mt genomes datasets entail 28 (4×7) initial alignments. Furthermore, both the SARS-CoV-2_20200301 and SARS-CoV-2_20200417 datasets each encompass 20 (4×5) initial alignments. The proportions of "improved", "constant", and "reduced" are calculated based on the number of initial alignments for each dataset. It’s worth noting that for the two SARS-CoV-2 datasets, ReformAlign’s results were not displayed due to exceeding the device’s memory limit.

Fig 1A and 1D demonstrate that the TPMA results consistently outperform the other nine initial alignments and the combined results of M-Coffee in terms of aSP, Q, and TC (except for the 99% similarity dataset) scores across the 16S-like and 23S-like rRNA datasets. This advantage becomes more pronounced as the dataset similarity decreases. For the 16S-like rRNA datasets with a 99% similarity, TPMA achieves an average TC score of 0.9967, while ClustalW2 and PCMA achieve an average TC score of 0.9970, and M-Coffee’s average TC score is 0.9957. Similarly, for the 23S-like rRNA datasets with a 99% similarity, TPMA attains an average TC score of 0.9963, while T-Coffee and M-Coffee achieve average TC scores of 0.9969 and 0.9960, respectively. Consequently, TPMA’s results closely approach the highest TC score and surpass those of M-Coffee. Furthermore, TPMA demonstrates shorter running time and lower memory consumption than M-Coffee on both sets of datasets (Fig 1B and 1E). In the 16S rRNA datasets with an average similarity of 75%, TPMA shows a superior aSP score compared to M-Coffee (Fig 2A). However, for the other three real datasets with high average similarities, TPMA and M-Coffee demonstrate close scores (Fig 2D, 2G, and 2J). Furthermore, TPMA exhibits a significantly shorter running time and consumes considerably less memory than M-Coffee (Fig 2B, 2E, 2H, and 2K).

Afterward, we assessed the performance of TPMA, M-Coffee, and ReformAlign in enhancing alignment quality. First, we optimized each initial alignment utilizing ReformAlign’s fundamental parameters (detailed in S10 Table), calculated the difference in aSP, Q, and TC scores between the optimized and initial alignments, and then computed the discrepancy between the combined results and each participating initial alignment for TPMA and M-Coffee.

On both 16S-like and 23S-like rRNA datasets (Fig 1C and 1F), TPMA improves or maintains the aSP score in all datasets, and it enhances Q and TC scores in the majority of datasets when compared to M-Coffee and ReformAlign. Similarly, compared to M-Coffee and ReformAlign, TPMA improves more alignments’ aSP score across all four real datasets, which were randomly sampled to obtain multiple replicas for comparison (Fig 2C, 2F, 2I, and 2L). It is noteworthy that ReformAlign faced limitations in optimizing the two SARS-CoV-2 datasets due to memory limitations exceeding the device’s upper threshold.

Thus, TPMA consistently imparts heightened alignment quality compared to most initial alignments, a trend especially pronounced in datasets exhibiting lower similarity. While, in certain instances, TPMA’s score might marginally fall below that of the initial alignment, it consistently approaches the highest score and surpasses the outcome achieved by M-Coffee. In contrast, the accuracy of M-Coffee generated alignments is susceptible to the accuracy of the initial alignment, potentially between the best and worst initial alignments, showing a limited ability to improve the quality of initial alignments. TPMA exhibits a shorter running time and less memory consumption than M-Coffee. Furthermore, as sequence length and quantity increase, ReformAlign’s memory demands become excessively high, constraining its scalability.

### Accurate strategy: achieving comparable results with 4 MSA tools as with All 9

**Fig 3:**
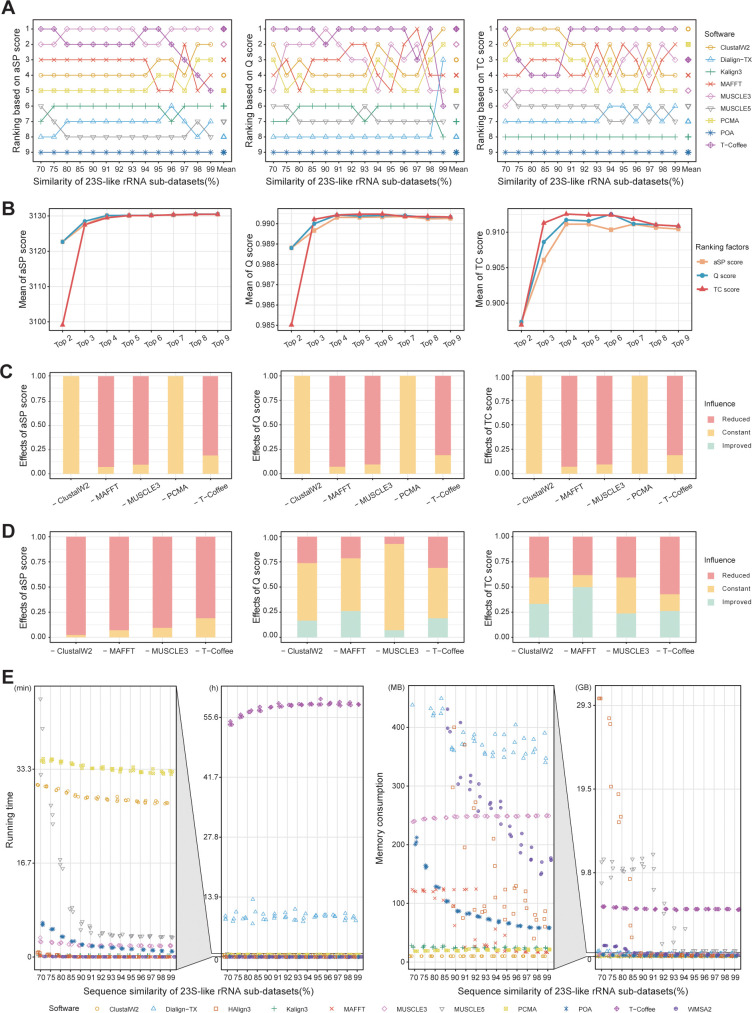
The results of screening the accurate strategy on the 23S-like rRNA datasets. **A** MSA tool rankings are determined based on aSP, Q, and TC scores. Each point corresponds to the average value of three replicates within that sub-dataset. "Mean" denotes the ranking derived from the average values spanning all sub-datasets with varying degrees of similarity. **B** Sequential integration of initial alignments from MSA tools following ranking by aSP, Q, and TC scores. The ranking is derived from the "Mean" results of **A**. Referring to the aSP score rankings (the orange line), the "Top 2" position indicates the integration of T-Coffee and MUSCLE3, while the "Top 3" placement corresponds to the fusion of T-Coffee, MUSCLE3, and MAFFT, and so forth. Analyzing the Q score rankings (the blue line), the "Top 2" position signifies the combination of T-Coffee and MUSCLE3, while the "Top 3" is the fusion of T-Coffee, MUSCLE3, and ClustalW2, and so forth. Similarly, based on the TC score ranking (the red line), the "Top 2" position indicates the combination of ClustalW2 and PCMA, while the "Top 3" placement represents the integration of ClustalW2, PCMA, and T-Coffee, and so forth. The outcomes represent the average values of the aSP, Q, and TC scores of combined alignments across all sub-datasets of different similarities. **C** Ablation experiment on the "Top 5" MSA tools. The disparities between the aSP, Q, and TC scores of the combined alignment obtained from the remaining four initial alignments (excluding the indicated MSA tool) and from all five initial alignments were computed. If the score from combining the remaining four initial alignments is higher, it’s classified as ’improved’; if it remains the same, it’s labeled ’constant’; if it decreases, it’s categorized as ’reduced’. Each histogram displays the proportions of "improved," "constant," and "reduced" cases, calculated from a total of 42 datasets (14*3) across all sub-datasets. **D** Ablation experiment on the "Top 4" MSA tools (similar to C). **E** The running time and memory consumption of 11 MSA tools on 42 23S-like rRNA datasets are presented. Logging was conducted using Python’s psutil library.

Initially, we arranged the 9 MSA tools based on the accuracy of their alignment results, revealing that the top 5 aligners derived from the aSP, Q, and TC scores were identical, comprising ClustalW2, MAFFT, MUSCLE3, PCMA, and T-Coffee, albeit in varying specific orders (Fig 3A). By utilizing MSA tools’ aSP score ranking, we added and merged the initial alignments one by one from the top-ranked MSA tools, then computed the average accuracy across all sub-datasets, illustrated as the yellow square curve (Fig 3B). "Top 2" signifies the result obtained by combining the alignments derived from the top two aligners, T-Coffee and MUSCLE3, by TPMA. "Top 3" represents the combined result of the top three T-Coffee, MUSCLE3, and MAFFT; the subsequent combinations are deduced accordingly. We replicate the identical procedure using the rankings derived from Q and TC scores, resulting in the outcomes represented by the blue circular curve and the red triangular curve, respectively. Notably, as more initial alignments were added, the aSP, Q, and TC scores initially increased but eventually reached a plateau or slightly decreased. This observation suggests that the inclusion of Dialign-TX, Kalign3, MUSCLE5, and POA in these combinations did not enhance the final alignment’s quality. Upon reanalysis of the aSP, Q, and TC scores across various sequence similarities (S1–S3 Figs), similar pronounced trends were observed in low-similarity sub-datasets, highlighting TPMA’s enhanced efficacy in integrating such datasets.

The trend from "Top 4" to "Top 5" remains constant, leading us to deduce the presence of redundant MSA tools within the "Top 5" combination. Consequently, we performed ablation experiments on the selected top 5 MSA tools, revealing that the final alignment remained unchanged when either ClustalW2 or PCMA was excluded (Fig 3C). Considering their running time and memory utilization on 23S-like rRNA datasets (Fig 3E), ClustalW2 demonstrates reduced memory consumption and faster processing time than PCMA. Consequently, we refined the accurate combination strategy to encompass four aligners: ClustalW2, MAFFT, MUSCLE3, and T-Coffee. Further, ablation experiments on these four MSA tools demonstrated that each aligner significantly contributed to the final alignment quality, affirming their indispensability within the accurate strategy (Fig 3D).

**Fig 4:**
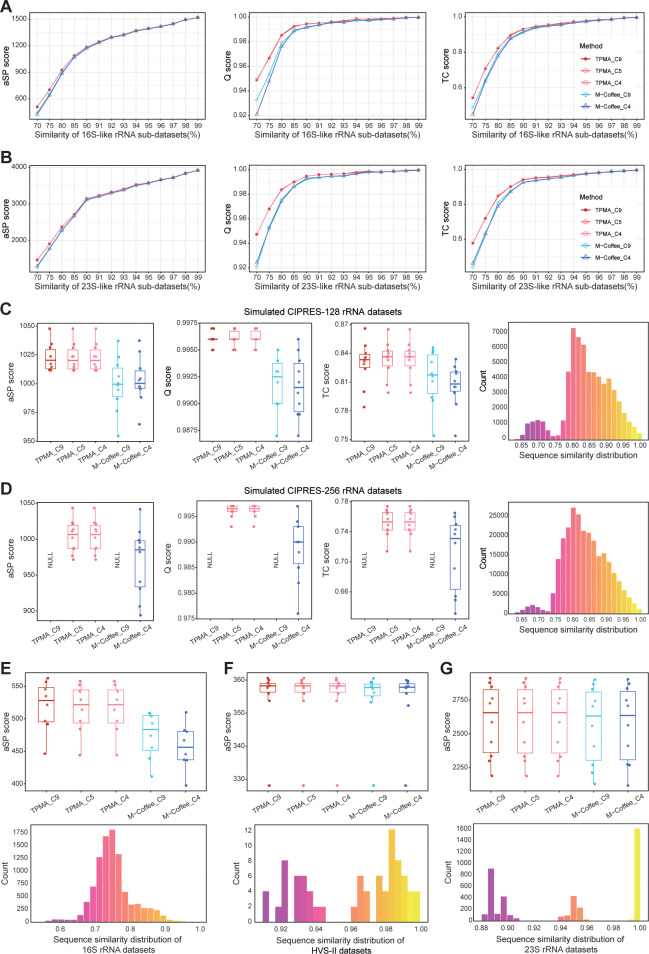
Validation of the accurate strategy on additional datasets. TPMA_C9 and M-Coffee_C9 represent the combined alignments of 9 initial alignments from ClustalW2, Dialign-TX, Kalign3, MAFFT, MUSCLE3, MUSCLE5, PCMA, POA, and T-Coffee. TPMA_C5 combines initial alignments from 5 MSA tools: ClustalW2, MAFFT, MUSCLE3, PCMA, and T-Coffee. TPMA_C4 and M-Coffee_C4 are the combined alignments of merging the initial alignments from the accurate strategy (ClustalW2, MAFFT, MUSCLE3, and T-Coffee). **A, B** We present the trends in aSP, Q, and TC scores of TPMA_C9, TPMA_C5, TPMA_C4, M-Coffee_C9, and M-Coffee_C4 on the 16S-like and 23S-like rRNA sub-datasets. The results represent the average value across the three replicates from each sub-dataset. **C, D,** Comparing aSP, Q, and TC scores (10 replicates) for TPMA_C9, TPMA_C5, TPMA_C4, M-Coffee_C9, and M-Coffee_C4 on simulated CIPRES-128 and CIPRES-256 rRNA datasets, alongside a sequence similarity distribution histogram. The sequence similarity of each replicate was similar within the dataset, and only one of the replicates’ sequence similarity distribution histograms was plotted. The "NULL" indicates that TPMA-C9 and M-Coffee_C9 produced no results due to the infinite alignment time of Dialign-TX. **E-G,** The aSP scores of TPMA_C9, TPMA_C5, TPMA_C4, M-Coffee_C9, and M-Coffee_C4 on the 16S rRNA, HVS-II, and 23S rRNA datasets with the corresponding sequence similarity distribution histograms (only one representative histogram was displayed). The 16S rRNA datasets have 8 replicates, while the HVS-II and 23S rRNA datasets contain 10 replicates.

We conducted tests on seven datasets to further validate the broad applicability of the accurate strategy when utilizing TPMA. TPMA combines nine initial alignments, "Top 5" initial alignments, and the four initial alignments from the accurate strategy to generate TPMA_C9, TPMA_C5, and TPMA_C4, respectively. In contrast, M-Coffee integrates the nine initial alignments and the four initial alignments of the accurate strategy, yielding M-Coffee_C9 and M-Coffee_C4, respectively. TPMA’s combined outcomes on 16S-like and 23S-like rRNA datasets consistently outperform M-Coffee in terms of aSP, Q, and TC scores. Meanwhile, TPMA_C9, TPMA_C5, and TPMA_C4 show highly similar results (Fig 4A and 4B). These three TPMA outcomes also demonstrate comparable performance across simulated CIPRES-128, CIPRES-256, and 16S rRNA datasets, where the sequences exhibit average similarity rates of 80% for the first two and 75% for the latter, all-surpassing M-Coffee (Fig 4C–4E). The "NULL" signifies that no results were acquired from TPMA-C9 and M-Coffee_C9 due to the infinite alignment time of Dialign-TX (Fig 4D). The HVS-II and 23S rRNA datasets exhibited an average similarity above 93%. Results from three TPMA and two M-Coffee alignments closely align on these datasets, with TPMA slightly surpassing M-Coffee’s outcomes (Fig 4F and 4G). Therefore, the outputs from the comprehensive test datasets validate that integrating the four alignments acquired through the accurate strategy can yield results of comparable quality to combining all nine alignments while saving time and memory.

### Fast strategy: enhancing alignment accuracy with time efficiency for large-scale datasets

During the above experiment, we observed that early-developed MSA software, such as T-Coffee and Dialign-TX, faced difficulties in aligning datasets with longer or larger sequences, leading to considerable time and memory expenses, potentially limiting options for certain users, particularly considering the inclusion of T-Coffee in the accurate strategy. To address this, we identify newly developed MSA software that exhibits swift alignment speed and lower memory consumption, and by integrating these tools with TPMA, we formulated a fast combination strategy, explicitly catered for datasets with larger scales, aiming to save time while preserving alignment accuracy as closely as possible to the accurate strategy results. We evaluated the performance of the fast strategy which selected the four most rapid MSA tools (Fig 3E), namely HAlign3, Kalign3, MAFFT, and WMSA2, by conducting experiments on eight datasets. The experimental approach involved running the four MSA tools to acquire initial alignments, followed by merging these alignments using TPMA to generate "TPMA_F4". We compared the accuracy of TPMA_F4 with that of "TPMA_C4" from the 4.2 experiment and recorded the memory consumption and running time required for obtaining all initial alignments. The memory consumption represents the maximum usage of TPMA_F4 and TPMA_C4 when running individual MSA tools, while the running time refers to the cumulative time taken by TPMA_F4 and TPMA_C4 when running each MSA tool. The time and memory consumption associated with combining initial alignments in TPMA_F4 and TPMA_C4 were disregarded.

**Fig 5:**
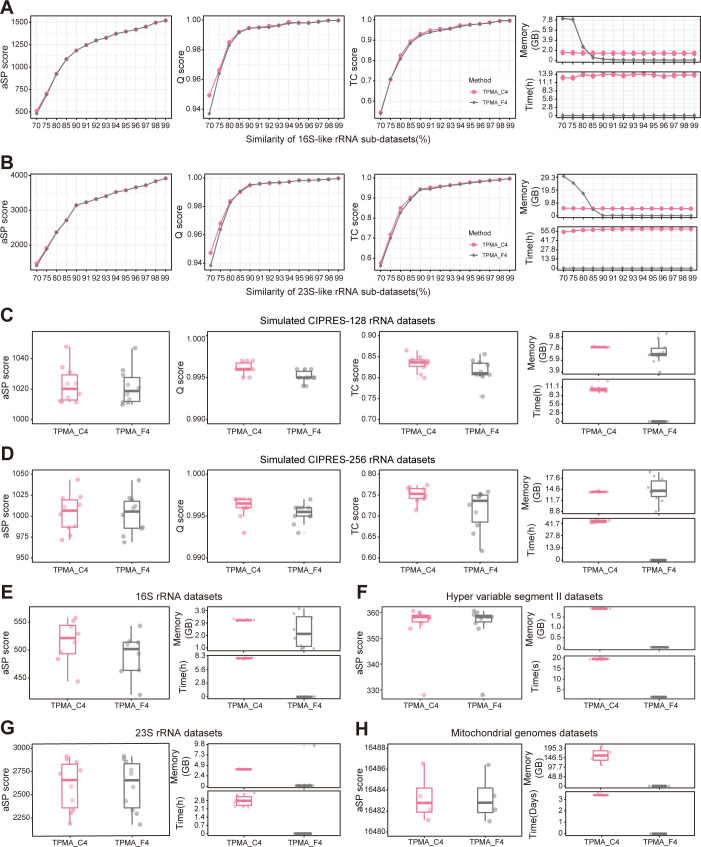
Comparative analysis of accurate and fast strategies. TPMA_C4 is derived from the merged alignment by combining the initial alignments obtained through accurate strategy (ClustalW2, MAFFT, MUSCLE3, and T-Coffee), while TPMA_F4 results from that of the fast strategy (HAlign3, Kalign3, MAFFT, and WMSA2). The time indicated the aggregate of the running time for the four MSA tools within the combined strategy. Meanwhile, the memory reflects the highest memory consumption observed during the aligning process of the four MSA tools in the combined strategy. **A-D** aSP, Q, and TC scores of TPMA_C4 and TPMA_F4 on the 16S-like, 23S-like, simulated CIPRES-128 and CIPRES-256 rRNA datasets, along with the overall time and memory peak consumption during acquiring all initial alignments. Each point in **A** and **B** represents the average value from three replicates within the sub-dataset of 16S-like and 23S-like rRNA datasets. Meanwhile, both CIPRES rRNA datasets consist of 10 replicates. **E-H** aSP score of TPMA_C4 and TPMA_F4 on the 16S rRNA, HVS-II, 23S rRNA, and mt genomes datasets with the time and memory of that required to obtain all initial alignments. The 16S rRNA, HVS-II, 23S rRNA, and mt genome datasets comprised 8, 10, 10, and 4 replicates, respectively.

In the 16S-like and 23S-like rRNA datasets, the aSP and TC score curves of TPMA_C4 and TPMA_F4 almost overlap, while, as the sequence similarity falls below 80%, the Q score of TPMA_F4 slightly lags behind that of TPMA_C4. Notably, TPMA_F4 demands significantly less time for initial alignments than TPMA_C4, and when the sequence similarity reaches 85% or higher, TPMA_F4 exhibits lower memory consumption than TPMA_C4 (Fig 5A and 5B). On the simulated CIPRES-128 and CIPRES-256 rRNA datasets (sequence similarity: 80%), the aSP score distributions are similar between the two methods, while the Q score and TC score distributions of TPMA_F4 are slightly lower than those of TPMA_C4 (Fig 5C and 5D). For the 16S rRNA datasets (sequence similarity: 75%), TPMA_F4 achieves a slightly lower aSP score than TPMA_C4 (Fig 5E). However, in the case of the HVS-II datasets (sequence similarity: 98.6%), 23S rRNA datasets (sequence similarity: 92.7%), and mt genomes datasets (sequence similarity: 99.7%), the TPMA_C4 and TPMA_F4 results demonstrate similar aSP score distributions (Fig 5F–5H). While the disparity in peak memory consumption between the two methods is minimal in the above datasets, TPMA_F4 requires significantly less time for initial alignments than TPMA_C4. Especially on the mt-genomes datasets, TPMA_C4’s completion time for all initial alignments extends up to 4 days, accompanied by memory usage exceeding 100GB, whereas TPMA_F4 accomplishes the task within a mere 1 minute, utilizing 290MB of memory. The experimental results affirm that, when sequence similarity exceeds 80%, the fast strategy can attain alignment quality comparable to the accurate strategy, significantly reducing the time required for initial alignments, particularly on extensive datasets.

## Discussion

We developed Two Pointers Meta-Alignment (TPMA) to enhance the quality of nucleic acid sequence alignments by combining blocks with high SP scores from multiple initial alignments. The assessment of TPMA across six real and four simulated datasets revealed that it improves the aSP, Q, and TC scores of initial alignments across most datasets, particularly in datasets with low sequence similarity, all while reducing memory and time consumption. While TPMA’s result exhibited a relatively lower score in partial high sequence similarity datasets, it closely approached the highest score of initial alignments and outperformed the result from M-Coffee. Furthermore, in contrast to M-Coffee and ReformAlign, TPMA demonstrates a more significant capability for enhancing the quality of initial alignments. Subsequently, we evaluated the MSA tools utilized in the experiment and presented users with accurate and fast strategies. Validation of the test datasets affirmed that these two strategies could uphold alignment accuracy while conserving time and facilitating the integration of large-scale datasets. In our experimental tests, we focused solely on comparing the running time and memory consumption of TPMA and M-Coffee, without including ReformAlign in the comparison. This decision stems from the fact that TPMA and M-Coffee employ similar optimization strategies, namely merging multiple initial alignments, while ReformAlign conducts re-alignment against a single initial alignment. As a result, a direct comparison with TPMA and M-Coffee in terms of time and memory consumption is not feasible.

During a validation experiment to assess TPMA’s efficacy, we employed 9 MSA tools for the integration. Recognizing the impracticality for users to execute numerous MSA tools individually before integration, we streamlined the process by offering users an accurate strategy, ensuring equivalent or similar alignment quality with the fewest tools. This enhancement in user convenience saves valuable time typically spent on tool selection. Additionally, 23S-like rRNA was chosen as the experimental dataset for screening MSA tools of accurate strategy for several reasons: firstly, the simulated datasets provide reference alignments, and we can comprehensively assess MSA tools based on aSP, Q, and TC scores. Among these metrics, aSP quantifies the quality of sequence alignment by computing the average similarity score across all sequence pairs. Q and TC provide a comprehensive assessment of alignment accuracy and completeness relative to a reference alignment. MSA tools that incorporate multiple indicators in their selection process tend to be more reliable. Secondly, compared with the simulated CIPRES datasets, its multiple sub-datasets with varying similarities allow for the identification of MSA tools less influenced by different similarities, ensuring broad applicability of the screening results. And finally, compared to the 16S-like rRNA datasets, 23S-like rRNA possesses longer sequence lengths and more noticeable disparities in running time and memory consumption among MSA tools, simplifying the decision-making process.

As sequencing technology has advanced, there has been a substantial increase in the length and quantity of sequences for a single alignment process. The mt genome, approximately 16 kb in length, and the SARS-CoV-2 genome, spanning around 29 kb, highlight this trend, with over 16 million SARS-CoV-2 genomes sequenced from 193 countries and regions as of July 2023. However, early-developed MSA tools face challenges when dealing with large-scale alignment tasks. For instance, T-Coffee and Dialign-TX experience infinite running times when aligning the SARS-CoV-2_20200301 sub-dataset containing 39 sequences. Although T-Coffee, included in the accurate strategy, delivers high-quality alignment outcomes, its performance is constrained when aligning extensive sequences, limiting the broad applicability of the accurate strategy. Hence, we opted for the newly developed tools HAlign3, Kalign3, MAFFT, and WMSA2 to create a fast strategy to produce initial alignments for large-scale datasets as quickly as possible. The experimental results demonstrate that the four MSA tools chosen via the fast strategy outperform those selected through the accurate strategy in terms of speed and suitability for large-scale data. Particularly when dealing with low sequence similarity, HAlign3 may exhibit high memory usage, but this limitation can be mitigated by reducing the number of parallel threads. Moreover, the decision to opt for four MSA tools in the fast strategy, rather than a smaller selection, stems from the advantage it offers in facilitating effective comparisons on large-scale datasets compared to the accurate strategy. A smaller tool selection could compromise the ability to make meaningful comparisons. Despite not being part of the accurate strategy, Kalign3’s rapid aligning and minimal memory usage on large-scale datasets align with the objectives of the fast strategy tailored for such datasets.

## Materials and methods

### The workflow of TPMA

**Fig 6:**
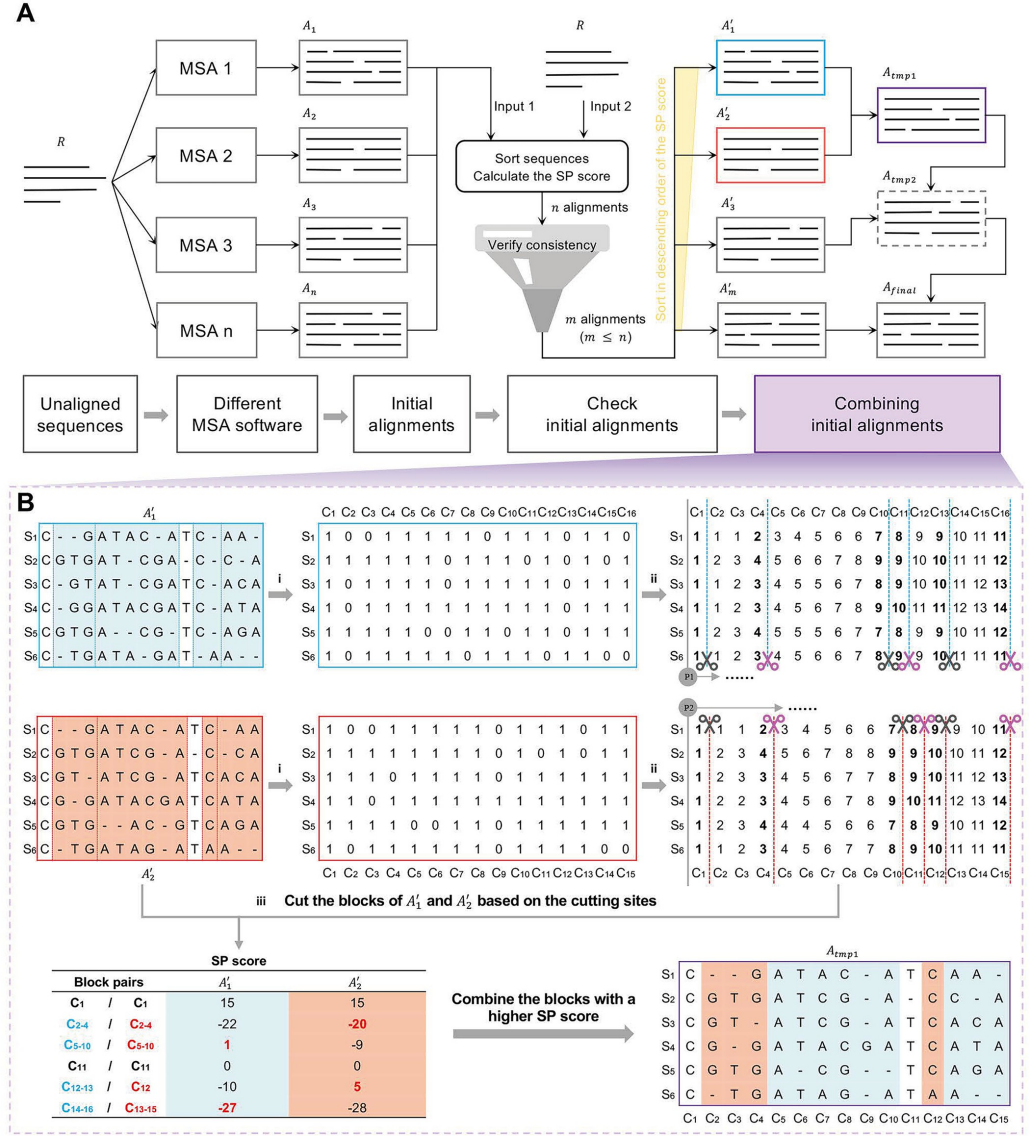
Operational mechanism and illustrative cases of TPMA. **A** Flowchart of TPMA. Unaligned sequences (*R*) are aligned using *n* MSA tools to generate *n* initial alignments, and these initial alignments along with the *R* are fed into TPMA. TPMA checks the *n* initial alignments by sorting sequences, calculating SP scores, and verifying for consistency, resulting in *m* valid initial alignments. The *m* valid initial alignments are combined in descending order of SP scores. A total of *m-1* combining steps are conducted to obtain the final alignment, denoted as *A*_*final*_. **B** Example of a detailed merging process for two initial alignments. (i) Recode $A_{1}^{\prime }$ and $A_{2}^{\prime }$ as binary strings consisting exclusively of 0s and 1s. (ii) $A_{1}^{\prime }$ and $A_{2}^{\prime }$ are divided into blocks according to the bold column *C*_*i*_ (*C*_1_, *C*_4_, *C*_10_, *C*_11_, *C*_13_ and *C*_16_ for $A_{1}^{\prime }$; *C*_1_, *C*_4_, *C*_10_, *C*_11_, *C*_12_ and *C*_15_ for $A_{2}^{\prime }$), which yields six pairs of blocks. Each of these block pairs consists of identical sequence fragments. (iii) Compute the SP scores for these blocks, and then merge the blocks with higher SP values into *A*_*tmp*1_. *A*_*tmp*1_ encompasses sequences of length 15, structured as follows: block *C*_1_ with identical scores, blocks *C*_2_−*C*_4_ from $A_{2}^{\prime }$, blocks *C*_5_−*C*_10_ from $A_{1}^{\prime }$, block *C*_11_ with the same score, block *C*_12_ from $A_{2}^{\prime }$, and finally, blocks *C*_13_−*C*_15_ from $A_{1}^{\prime }$.

TPMA requires two inputs: firstly, a set containing *A*_1_, *A*_2_,…,*A*_*n*_ (the initial alignments of the original sequence dataset *R* by *n* MSA tools) and secondly, the original unaligned sequence dataset *R*. The default output sequence order of MUSCLE3, MUSCLE5, and PCMA is based on the clustering of their guide tree, which often differs from the sequence order of the original dataset. Furthermore, when the original sequences contain characters other than the 26 uppercase and lowercase English letters, MAFFT and MUSCLE3 will align the sequences after deleting these characters. Therefore, TPMA mandates a meticulous examination of the initial alignments (Fig 6A).

Firstly, TPMA reorders the sequences in each initial alignment *A*_*i*_ to match the order of the original dataset *R* and computes the sum of pairs (SP) score for each reordered alignment $A_{i}^{\prime }$. Subsequently, the characters except for the 26 uppercase and lowercase English letters are removed from the sequences in $A_{i}^{\prime }$ and *R*, and then the consistency of each $A_{i}^{\prime }$ with *R* is checked. The alignments that differ from *R* will be deleted. In the end, we acquire a subset of validated initial alignments, denoted as $A_{1}^{\prime },A_{2}^{\prime },\ldots ,A_{m}^{\prime }$, where *m*≤*n*.

Subsequently, we merged all validated initial alignments in descending order according to the SP score. We first combine the two alignments $A_{1}^{\prime }$ and $A_{2}^{\prime }$ with the highest SP scores to get *A*_*tmp*1_, then merge *A*_*tmp*1_ and $A_{3}^{\prime }$ with the third highest SP score to obtain *A*_*tmp*2_, and continue this process a total of *m*−1 times to generate the final alignment *A*_*final*_. *A*_*final*_ combines blocks with higher SP scores from the initial alignments, resulting in a refined alignment (Fig 6A.)

### The combination of two initial alignments

Our research focuses on partitioning two initial alignments into block pairs with the same sequence fragments, as depicted in Fig 6B, including the following three steps:

### Recode the initial alignments

After checking the initial alignments, the sequences in all the remaining alignments are in the same order but probably inserted with gaps in different positions. TPMA first recodes the remaining alignments by mapping gaps to 0 and all the other characters to 1. This recoding ensures that the base composition of each sequence becomes irrelevant, facilitating the identification of gap insertion positions. As shown in Fig 6B_i_, TPMA recodes $A_{1}^{\prime }$ and $A_{2}^{\prime }$ to obtain binary strings consisting of only 0s and 1s.

### Find the cutting sites

Two pointers, *P*_1_ and *P*_2_, independently traverse their respective recoded alignments and simultaneously record their corresponding "pace," denoted by *pace*_1_ and *pace*_2_. The “pace” is an array that records the cumulative count of "1"s scanned in each sequence up to the current column. (Fig 6B_ii_). *position*_1_ and *position*_2_ respectively, keep track of the current column in the alignments where pointers *P*_1_ and *P*_2_ are located. Initializing the arrays *pace*_1_ and *pace*_2_, both with length is the number of sequences, to zero, and setting *position*_1_ and *position*_2_ to 0 indicates that the two pointers are at column 0 and in their initial state.

Move a pointer arbitrarily. If *P*_1_ scans first, when the pace of *P*_1_ is greater (any number in *pace*_1_ is greater than the corresponding number in *pace*_2_ in the same index) than that of *P*_2_, *P*_1_ stops scanning and *P*_2_ starts to scan. Similarly, if the pace of *P*_2_ becomes greater than that of *P*_1_, *P*_2_ stops scanning, and *P*_1_ continues to scan. Repeat this process alternately until both pointers (*P*_1_ and *P*_2_) have scanned all the columns in their respective alignments. When the pace is the same, the current columns (*position*_1_ and *position*_2_) of *P*_1_ and *P*_2_ represent the cutting sites corresponding to the two alignments (the columns with the bold number in Fig 6B_ii_). Assuming the sequence lengths of recoded *Seq*_1_ and *Seq*_2_ are n and m respectively, where *n* is less than *m*, the time complexity of completing the finding cutting point algorithm is *O*(*max*(*n*, *m*)), specifically *O*(*n*). The pseudocode for finding cutting sites is shown in Algorithm 1.

**Algorithm 1** Find the cutting sites

**Input:** two recoded alignments *Seq*_1_ and *Seq*_2_, both are DNA or RNA sequences with the same sequence order.

**Output:** two lists of cutting sites *L*_1_ and *L*_2_

**Function** CUTTING_SITES (*Seq*_1_, *Seq*_2_)

 Let *L*_1_ and *L*_2_ be the new lists

Let *P*_1_ and *P*_2_ be the two pointers of *Seq*_1_ and *Seq*_2_, respectively

Let *pace*_1_ and *pace*_2_ be the recording of the “pace” of *P*_1_ and *P*_2_,

Let *position*_1_ and *position*_2_ be the recording of the current positions of *P*_1_ and *P*_2_, respectively

Let *col*_1_ be the columns count of *Seq*_1_, *col*_2_ be the columns count of *Seq*_2_

 **do{**

  **for (*position*_1_! = *col*_1_ and** *pace*_2_<*pace*_1_ **= = FALSE)**

  **if (++***pace*_1_ = = *pace*_2_**)**

    *L*_1_.puch_back(*position*_1_)

    *L*_2_.puch_back(*position*_2_)

   **end if**

   **end for**

  **for (*position*_2_! = *col*_2_ and** *pace*_1_<*pace*_2_ **= = FALSE)**

  **if (++** *pace*_2_ = = *pace*_1_**)**

    *L*_1_.puch_back(*position*_1_)

    *L*_2_.puch_back(*position*_2_)

   **end if**

   **end for**

  **}while (*position*_1_! = *col*_1_** or ***position*_2_! = *col*_2_)**

  **return *L*_1_** and *L*_2_

**end Function**

### Choose superior blocks based on the SP score

According to all the identified cutting sites, the two initial alignments are partitioned into an equal number of blocks, and the blocks containing identical sequence fragments are referred to as one block pair. After calculating the SP score of each block pair, the block with the higher SP score is merged into the combined alignment (Fig 6B_iii_). The SP score is calculated as shown in Eq 1.


$$SP=\sum_{k=0}^{L}f(k)$$ (1)

The SP score is obtained by summing the scores of the *L* columns, where *f*(*k*) represents the score of the *k*-th column, and *L* denotes the total number of columns in the block. The calculation method for *f*(*k*) is shown in Eq 2.


$$f(k)=\sum_{i}^{N}\sum_{j\neq i}^{N}score(i,j)$$ (2)

Here, *N* is the number of sequences in the block, and *score*(*i*, *j*) represents the score of aligned pair in the *i*-th row and *j*-th row. If the aligned pair is matched (both characters are letters and they are the same), a score of 1 is assigned. For mismatched pairs (both characters are letters but different), a score of -1 is given. When one character is a letter and the other is a gap, a score of -2 is provided. Otherwise, a score of 0 is assigned.

### Datasets

The experiments utilized ten nucleotide datasets, comprising six real datasets and four simulated datasets (S1 Table). Challenges arose for certain Multiple Sequence Alignment (MSA) tools, such as Dialign-TX [13] and T-Coffee [14], in aligning datasets with extended sequence lengths or numerous sequences. We conducted multiple random samplings or partitioned the real dataset to reduce its size, generating multiple sub-datasets as test datasets. This approach helped eliminate the randomness and variability of the experimental results.

The six real datasets consist of five DNA datasets and one RNA dataset. (1) The human mitochondrial (mt) genomes dataset comprised 672 human mitochondrial genomes, ranging from a maximum length of 16579 bp to a minimum of 16556 bp [15]. We created sub-datasets by randomly selecting 30 sequences without replacement, and this procedure was repeated four times (S2 Table). (2) The hyper-variable segment II (HVS-II) dataset consisted of 100 sequences extracted from the HVS-II control region of the central European human mitochondrial genomes [16], retrieved from the GenBank database (accession numbers: KF601094-KF601193). The dataset was subsequently partitioned into ten sub-datasets (S3 Table). (3) The 16S ribosomal RNA (rRNA) dataset encompassed 108,413 DNA sequences encoding RNA found in bacteria and archaea, with an approximate length of 1.5 kb [17]. We created eight sub-datasets for this dataset, each containing 100 sequences sampled randomly without replacement (S4 Table). (4) The 23S rRNA dataset encompassed 641 sequences of Mycobacterium 23S rRNA, sourced from the SILVA rRNA database (http://www.arb-silva.de/) for bacteria, archaea, and eukaryotes. Spanning lengths from 1909 to 3485 bp, these sequences were partitioned into ten groups (S5 Table). (5) The respiratory syndrome coronavirus 2 (SARS-CoV-2) is an RNA virus that causes the COVID-19 pandemic. Two datasets [18] were derived from the GISAID website (https://www.gisaid.org, updated November 11, 2021.) The SARS-CoV-2_20200301 datasets contain 156 sequences (29409 to 29927 bp) collected on March 1, 2020, and were divided into four sub-datasets (S6 Table). (6) The SARS-CoV-2_20200417 datasets feature 1020 sequences (29409 to 29927 bp) collected on April 17, 2020. From this, we randomly selected 100 sequences without replacement to create a sub-dataset, and this process was repeated four times (S7 Table).

Two of the four simulated datasets were generated using hierarchical tree simulation to obtain 16S-like and 23S-like rRNA datasets. This simulation was carried out using INDELible v1.03 [19], and the substitution models were based on estimates obtained from 3000 16S rRNA and 641 Mycobacterium 23S rRNA alignments (as previously mentioned), utilizing IQ-TREE v2.2.0-beta [20]. One hundred 16S rRNA sequences and Mycobacterium 23S rRNA sequences, randomly chosen from the datasets as mentioned above, were aligned to construct the simulation trees. Subsequently, the process of generating the simulation 16S-like and 23S-like datasets was rooted in these two simulation trees. Each tree’s branch length was assigned a random value from 0 to 1 (NON-ULTRAMETRIC). The simulated sequence lengths were set at 1.5 kb for 16S-like rRNA and 4 kb for 23S-like rRNA. The indel model parameter used was LAV 5 50, with insertion and deletion rates of 0.01 and 0.1, respectively. To simulate datasets with varying mean similarities, the tree length (sum of branch lengths) was adjusted to achieve mean similarities of 99%, 98%, 97%, 96%, 95%, 94%, 93%, 92%, 91%, 90%, 85%, 80%, 75%, and 70%. The mean similarity represents the average of similarities between any two sequences within the dataset (https://github.com/malabz/MSATOOLS/blob/main/length_similarity_distribution/length_similarity_distribution.py), determined by the percentage of matched characters in their pairwise alignments conducted using MAFFT. Detailed information regarding these two sets of 14 sub-datasets can be found in S8 Table. Every sub-dataset included three replicates, each containing 100 simulated sequences. The mean length is the average of the average sequence lengths from the three replicates.

The remaining two datasets are simulated CIPRES-128 and CIPRES-256 rRNA datasets evolved from the same root rRNA sequence on the trees featuring 128 and 256 taxa, respectively (S9 Table). These datasets were downloaded from trials 1 to 10 on the CIPRES SIMULATION DATA website (https://kim.bio.upenn.edu/software/csd.shtml). The simulation parameters were adjusted to ensure that the simulated sequences mirror authentic small subunit rRNA (ssu rRNA) sequences regarding sequence identity, indel count, the ratio between substitutions and indels, and other relevant characteristics. The datasets were formatted in NEXUS format. The Nexus to Fasta Sequence Convert tool (http://www.bugaco.com) was utilized for conversion into FASTA format to obtain the reference sequences. Subsequently, gaps were removed from the reference to derive the unaligned test datasets.

Sequence similarity is computed for every dataset as the ratio of matching base pairs in all pairwise alignments (generated using MAFFT) within a sub-dataset. The script for this computation is available on https://github.com/malabz/MSATOOLS/blob/main/length_similarity_distribution/length_similarity_distribution.py. Lastly, employ Python’s Matplotlib library to generate a histogram depicting the distribution of sequence similarity.

### Software versions and operating parameters involved in this study

Furthermore, the experiment involved initial alignments obtained from eleven MSA tools: ClustalW2 [21], Dialign-TX, HAlign3 [22], Kalign3 [23], MAFFT, MUSCLE3, MUSCLE5 [24], PCMA [25], POA[26], T-Coffee, and WMSA2[27]. The identical software versions and running commands were used for obtaining initial alignments by TPMA and M-Coffee, with detailed information provided in S10 Table. In addition, the running commands of TPMA and M-Coffee merging the initial alignment from different MSA tools are shown in S11 Table.

### Evaluation and experimental environment

When evaluating alignment quality for the six real datasets, we employed the average sum of pairs (SP) score, denoted as aSP score, which represents the SP score divided by the number of sequence pairs, for accuracy assessment. The penalty parameters remained consistent with those detailed in the Methods 4.2.3 section. Regarding the four simulated datasets, in addition to the aSP score, we incorporated the Q score (the number of correctly aligned base pairs divided by the aligned pairs in the reference) and the TC score (the number of correctly aligned columns divided by aligned columns in the reference), both computed using the qscore program (http://www.drive5.com/bench/), to gauge the deviation between alignment outcomes and reference alignments. All experiments were conducted on a server running the Ubuntu Linux operating system, equipped with an Intel(R) Xeon(R) Platinum 8168 CPU operating at a clock speed of 2.7 GHz and 1TB RAM.

## Supporting information

S1 Fig The aSP score of the combination by sequential merging of initial alignments from MSA tools based on ranking by aSP, Q, and TC scores. The 14 subplots illustrate the results of 23S-like rRNA sub- datasets with different similarities: 99%, 98%, 97%, 96%, 95%, 94%, 93%, 92%, 91%, 90%, 85%, 80%, 75%, 70%. The points on each subplot represent the averages of three replicates. The aSP score-based ranking (the orange line) reveals that the Top 2 combination consists of T-Coffee and MUSCLE3, followed by the Top 3 combination of T-Coffee, MUSCLE3, and MAFFT, and so forth for subsequent combinations. Regarding Q scores ranking (the blue line), the Top 2 combination features T-Coffee and MUSCLE3, followed by the Top 3 combination of T-Coffee, MUSCLE3, and ClustalW2, and so on. When ranked by TC scores (the red line), the Top 2 combination consists of ClustalW2 and PCMA, with the Top 3 being ClustalW2, PCMA, and T-Coffee, and so forth.
(EPS)

S2 Fig The Q score of the combination by sequential merging of initial alignments from MSA tools based on ranking by aSP, Q, and TC scores. The plots depict the Q scores obtained by merging different initial alignment combinations across 14 23S-like rRNA sub-datasets. Each point displays the average of three replicates within the sub-dataset. The merged process is the same as described in. S1 Fig legend.
(EPS)

S3 Fig The TC score of the combination by sequential merging of initial alignments from MSA tools based on ranking by average SP, Q, and TC scores. The TC scores from combining various initial alignment combinations across the 23S-like rRNA sub-datasets are shown in the 14 subplots. These sub-datasets exhibit different similarities: 99%, 98%, 97%, 96%, 95%, 94%, 93%, 92%, 91%, 90%, 85%, 80%, 75%, 70%. All points on each subplot are the averages of three replicates. The merging process follows the same steps as described in S1 Fig legend.
(EPS)

S1 Table The summary of datasets. (XLSX)

S2 Table The detailed information of human mitochondrial genomes sub-datasets. (XLSX)

S3 Table The detailed information of human mitochondrial HVS-II sub-datasets. (XLSX)

S4 Table The detailed information of 16S rRNA sub-datasets. (XLSX)

S5 Table The detailed information of 23S rRNA sub-datasets. (XLSX)

S6 Table The detailed information of SARS-CoV-2_20200301 sub-datasets. (XLSX)

S7 Table The detailed information of SARS-CoV-2_20200417 sub-datasets. (XLSX)

S8 Table The detailed information of 16S-like and 23S-like rRNA of 14 sub-datasets. (XLSX)

S9 Table The detailed information of simulated CIPRES-128 and CIPRES-256 rRNA sub-datasets. (XLSX)

S10 Table Version information and operating parameters of the involved tools. (XLSX)

S11 Table The running command of TPMA and M-Coffee. (XLSX)
